# The cerebellum-driven social basis of mathematics: implications for one-on-one tutoring of children with mathematics learning disabilities

**DOI:** 10.1186/s40673-021-00136-2

**Published:** 2021-05-10

**Authors:** Larry Vandervert, Kimberly Moe

**Affiliations:** 1 American Nonlinear Systems, Spokane, USA; 2 Dept. of Education, Adjunct, Whitworth University, Spokane, USA

**Keywords:** Arithmetic tutoring, Autobiographical knowledge, Cerebellum, Dyscalculia, Mathematics, Mentalizing, One-on-one tutoring, Phonological processing, Stone-tool making, Theory of mind

## Abstract

The purpose of this article is to argue that the patterns of sequence control over kinematics (movements) and dynamics (forces) which evolved in phonological processing in inner speech during the evolution of the social-cognitive capacities behind stone-tool making that led to the emergence of *Homo sapiens* are homologous to the social cerebellum’s capacity to learn patterns of sequence within language that we refer to as mathematics. It is argued that this evolution (1) selected toward a social cognitive cerebellum which arose from the arduous, repetitive precision patterns of knapping (stone shaping) and (2) that over a period of a million-plus years was selected from mentalizing toward the kinematics and dynamics as observed and modeled in Theory of Mind (ToM) of more experienced stone knappers. It is concluded that components of this socially-induced autobiographical knowledge, namely, (1) segmenting events, (2) sequencing events, and (3) sequencing event clusters, all at various levels of abstraction, can inform optimum approaches to one-on-one tutoring of children with mathematical learning disabilities.

## Introduction

Based on the works of Ito [1–4], Leiner, Leiner and Dow [5, 6], Leggio and Molinari [7], Van Overwalle and Mariën [8] and Van Overwalle et al. [9], Vandervert [10–12] proposed that the foundations of our scientific knowledge of patterns that constitutes mathematics^1^ evolved in the human brain during approximately 1.7 million years of rigorous, repetitive social interaction required in stone-tool making. Within this theoretical perspective he argued that the patterns which constitute mathematics (1) evolved in cerebellar internal models of repetitive *patterns of sequences* (a la [7]) of detailed cause-and-effect related kinematics and dynamics of socially modeled stone-tool making [12] and (2) evolved as the basis for mentalized forms of knowledge [within Theory of Mind (ToM)]through the phonological loop of inner speech representing the socially modeled kinematics and dynamics of other bodies in (1). That is, the capacity for a knowledge of the patterns of mathematics evolved in inner speech as manipulated within ToM (one’s simulative capacity of make automatic and intuitive anticipatory inferences about the mental states of others) ([9, 13–16]; Van Overwalle & Mariën, 2015). It is important to note in regard to this second argument that following Baddeley et al. [17] the phonological loop evolved in working memory primarily to learn new word forms—more will be said of the evolution of the phonological loop in a moment.

## Purpose

The purpose of this article is threefold. First, it is argued that (1) the cerebellum’s phonological patterning of sequences representing the social kinematics and dynamics of stone-tool making and (2) those which the social cerebellum learns as the basis for the patterns that come to constitute mathematics in each child (see footnote-1) are *homologous* (share common evolutionary descent). Second, within this perspective, it is further argued that the learning of mathematics, like the learning of stone-tool making, takes place predominately in cerebellar internal models of inner speech in the child that are based on patterns of the kinematics and dynamics of social interaction as modeled in Theories of Mind (ToM) of the teacher.^2^ Third, it is therefore proposed that the efficacy of one-on-one teaching/learning of mathematics (e.g., [19]) is predominantly (1) based on the learning of constantly new phonological representations (new words) in inner speech, and (2) this phonological process can be optimized in children with mathematics learning disabilities (MLDs) including dyscalculia when taught with an emphasis on (ToM) autobiographical components identified by Van Overwalle et al. [9].

## Stone-tool making and the million-plus years social evolution of cerebellar internal models of patterns of sequences underlying mathematics

To fully understand the foregoing arguments for a prominent role of the cerebellum in the foundations of mathematics, it is important for the reader to be acquainted with pertinent details of the million-plus years of socially mediated kinematics (movements) and dynamics (forces) challenges and resulting adaptive selection during stone-tool evolution. Thus, neuro-anthropologists Stout and Hecht’s [20] somewhat detailed description of the rigorous, repetitive stone-tool knapping (stone shaping) process is quoted at length:

Knapping is a “reductive” technology involving the sequential detachment of flakes from a stone core using precise ballistic strikes with a handheld hammer (typically stone, bone, or antler) to initiate controlled and predictable fracture [such prediction would have *required the acquisition of internal primitive intuitive knowledge or mentalizing of kinematics and dynamics of cause-and-effect relationships*]. This means that small errors in strike execution can have catastrophic, unreversible effects. Experiments by Bril and colleagues have shown that fracture prediction and control is a demanding perceptual-motor skill reliably expressed only in expert knappers (Nonaka, Bril & Rein, 2010 [21];).

The key bottleneck in the social reproduction of knapping is thus the extended practice required to achieve perceptual-motor competence. This requires mastery of relationships, for example between the force and location of the strike and the morphology, positioning, and support of the core [22–24] [such mastery would *require neural coding of detailed kinematics and dynamics of strikes related to predictive fractures*], that are not perceptually available to naïve observers and cannot be directly communicated as semantic knowledge. Attempts to implement semantic knowledge of knapping strategies before perceptual motor skill development are ineffective at best [25, 26], and such knowledge decays rapidly along knapping transmission chains when practice time is limited, even if explicit verbal teaching is allowed [27]. For *observational learning* [italic added], the challenge is to translate visual and auditory information of another’s actions to appropriate motor commands for one’s own body. This may be accomplished by linking the observed behavior with preexisting internal models [the authors here are referring to models in the cerebral cortex, not in the cerebellum] of one’s own body and actions through associative learning and stimulus generalization ([28]; Laland & Bateson 2001) …. These learning challenges call for an interactive approach that alternates social-learning opportunities (observation, instruction) with motivated individual practice [29], as commonly seen in coaching and apprenticeship practice. (p. 7862–7863).

## In the last million-plus years, the cerebellum became prominent in driving the evolution of uniquely human pattern-based social-cognitive autobiographical components

Vandervert [11, 13] pointed out that in Stout and Hecht’s [20] above description and analysis of the evolution of stone-tool knapping and the brain, they concentrated only on functions of the cerebral cortex. This is an important shortcoming of their approach for two reasons. First, there is an abundant amount of research now available (cited in brief at the beginning of this article) on how functions of the cerebellum (both motor and nonmotor) are brought into play by repetitive activities. These cerebellar functions can help provide detail on how knapping is learned in the brain and, thereby, shed additional light on fine details of how stone-tool making evolved. Second, and more directly addressing the parallels between stone-tool making and the learning of mathematics, research demonstrates that the cerebellum provides the basis for learning *pattern detection* [7] that can lead to the control of a precise level of knapping strike requirements. This precision in pattern learning, according to Stout and Hecht, must be observed in the detailed kinematics and dynamics of the body of the teacher and translated to the learner’s own body. Further, Vandervert [11, 13] observed that Stout and Hecht’s knapping scenario likely constituted social cognitive processing in the cerebellum that produced progressively more detailed inner speech in the learner’s working memory that (in both ontogeny and phylogeny) enabled a progressively more detailed, automatic and intuitive Theory of Mind (ToM) of the teacher. Due to its foregoing sequential nature in the adaptive formation of verbal working memory over a million-plus years of stone-tool evolution, this constituted a cerebellum-driven patterned autobiographical knowledge, the record of representations of one’s own life experiences that is automatically retrievable into working memory at different levels of abstraction and time scales. This scenario is strongly supported by Brozzoli et al. [30] who argued that increases in complexity during stone-tool evolution preceded increases in complexity in language evolution.

## Social cerebellar modeling of knapping strike patterns in stone-tool making and cerebellar modeling of patterns in mathematics are homologous: composition, decomposition and blending of cerebellar internal models

Based on the foregoing, it can reasonably be argued that the cerebellum builds constantly new socially-mediated internal models in phonological processes of working memory that apply not only to patterns of sequences (a la [7]) in observed movements (kinematics) and forces (dynamics) of stone-tool making, but across *all* behavioral and mentalizing processes (in ToM). It is proposed that this is so because inner speech apparently evolved within the context of stone-tool making, the patterns derived from any and all subsequent new phonological processing in inner speech must therefore comport with internal models from stone-tool making kinematics and dynamics-related components of mentalizing; otherwise, cause-and-effect relationships could not apply to abstract thoughts, which, themselves to be predictive in imaginary scenarios would have to be modeled across such kinematics and dynamics-related components of mentalizing in the first place. More will be said on the evolution of inner speech and abstract thought and the components of mentalizing in a moment. This process, it is suggested, produced/es the capacity for a conscious manipulation in working memory areas of the cerebral cortex of a “cerebellum-driven science of patterns” across all phenomena—in other words a capacity for a knowledge of mathematics (see footnote-1). Stone-tool evolution, by building a social cerebellum with inner speech, built a brain that could consciously think about abstract entities.

Vandervert [11, 31] argued that, in both ontogeny and phylogeny, the cerebellum accomplishes this “universal generalization” toward new representations of movements and forces through the composition, decomposition and blending of cerebellar internal models [32–35] of inner speech in phonological processing in working memory. It is through these cerebellar mechanisms (composition, decomposition, blending), it is further suggested, that, as proposed by Baddeley et al. [17], the phonological loop in working memory evolved to produce constantly new word forms. That is, cerebellar blending offers a deeper mechanism that could explain how in early human evolution new words “creatively” emerged within conscious inner speech the first place.^3^ In abstract conscious inner speech-driven imagination, these processes of composition, decomposition and blending of cerebellar internal models turned stones into progressively more efficiently predictive cause-and-effect instruments. Placed in Stout and Hecht’s [20] earlier-quoted description of stone-tool making these new words would have represented newly modeled (in cerebellar internal models) predictive kinematics and dynamics of the actions of other persons. This central role of inner speech within (1) the evolution of stone-tool making as the basis of new cognitive landscapes of abstract thought in relation to predictive patterns of sequences, and (2) the evolution of language is strongly supported by Crespi et al. [38, 39] recent findings on roles of the FOXP2 gene in language evolution:

To the extent that the adaptive amino acid evolution of FOXP2, along the human lineage, affected phenotypes comparable to those implicated here, *our results would suggest that inner speech played an important role in the origin and evolution of human language* [italics added]. This is an interesting hypothesis given evidence for causal connections of inner speech with abstract thought, cognitive performance, aspects of learning and development, and default-mode network mental functions [40–42]. (p. 38).

## Optimizing one-on-one mathematics teaching

Based on the prominent social role of the cerebellum in (1) one-on-one rigorous, repetitive social learning of the patterns of kinematics and dynamics of stone-tool making and (2) at the same time, providing the patterns underlying mathematics, a purpose of this article is to describe how difficulty in the learning of arithmetic (including dyscalculia) can be reduced by teaching young children in socially enhanced one-on-one frameworks that are based on the social-cognitive functions of the cerebellum.

It is commonly found that one-on-one tutoring improves the learning of arithmetic in young children [43–45]. Essentially, these studies are attempts to ascertain the effectiveness of various one-on-one tutoring approaches in producing what Dehaene [46] referred to as “number sense” (fluidity or automaticity in understanding and manipulating numbers). Vandervert [10] described the role of cerebellar inverse dynamics internal models, which through practice was predominant in producing this number sense fluidity in both understanding and manipulation of numbers in inner speech.

Along this line, Iuculano et al. [19], for example, found that children with mathematical learning disabilities (MLD) can improve significantly through an intensive eight-week course of one-on-one training with a tutor. This training of course was highly social and contained a good deal of repetition of number manipulation. Through this training, the MLDs became less overengaged in disparate areas of the cerebral cortex (notably, the prefrontal, parietal, ventral temporal–occipital cortices), and thus their brains became more like those of control subjects. However, in their conclusions Iuculano et al. did not mention the fact that their intense one-on-one training was found to engage bilateral areas of the cerebellum. Since the one-on-one tutorial training involved repetitive one-on-on tutoring and a high level of intense prolonged practice which, together, likely employed intense social-cognitive mentalizing learned in cerebellar internal models [8, 9, 47] it was highly probable that new or enhanced cerebellar connections with the diverse areas of the cerebral cortex were key to the corrective alignment of activity of the brain’s overengagement in the MLDs. That is, hypothetically, newly learned cerebellar forward anticipatory control of patterns of sequence through higher-level mentalizing (in phonological processing in inner speech) [7, 48] would reduce overengagement in wide areas of the cerebral cortex.

This idea is strongly supported by three lines of cerebellum research. First, it is now established that through their optimization and automaticity, the role of cerebellar internal models is to bring specific skill-related areas of the cerebral cortex under efficient goal-related anticipatory control ([48]; Bostan, Dum & Strick, 2003 [3];). Second, in social learning, the cerebellum learns internal models of the behavior and imagined thoughts of others—these are referred to as theory of mind (ToM) models [9, 13, 15, 49]. Third, it provides for new word-induced mentalizing about number through inner speech via the phonological loop in verbal working memory [17], which then becomes automatic and fluid (number sense) through the repetition component.

## The components of the Child’s Mentalizing during one-on-one tutoring in arithmetic

Specially, it is proposed that as in social learning as hypothesized by Van Overwalle et al. [9], the learning of arithmetic in young children occurs, as described earlier in this article, in the context of socially driven development of inner speech in the phonological loop of verbal working memory [15]. The role of phonological processing in early arithmetic learning is strongly supported by, for example, Simmons and Singleton [50] and Soto-Calvo et al. [51]. It is further proposed that this new inner speech learning would have occurred within autobiographical mentalizing (judgments about past and future personal events) [9] within the one-on-one tutoring of the child. For arithmetic (or any mathematical patterns) this autobiographical mentalizing would include at least (1) segmenting events, (2) sequencing events, and (3) sequencing event clusters *at various levels of abstraction* as described by Van Overwalle et al. Within the framework presented in this article which proposes a homological relationship between the sequence patterning/inner speech cerebellum that evolved in stone-tool evolution and the patterning that constitutes mathematics, the abstract autobiographical mentalizing required in learning stone-tool making would have (1) shifted toward progressively higher levels of abstraction in the construction of later Acheulean tools about 1.5 million years ago [52], and (2) subsequently toward the highest levels existent levels of abstraction in mathematics. The evolution of this autobiographical mentalizing scenario presents another way of understanding the origins of the social basis of mathematics.

The cerebellum-driven autobiographical conception of arithmetic learning also leads to a way to better understand and optimize the efficiency of a one-on-one social cognitive tutoring process. Specifically, Van Overwalle et al. [9] hypothesized how the cerebellum contributes to the process of making what is learned in such autobiographical knowledge automatic and intuitive:

We hypothesize that the cerebellum acts as a “forward controller” of social, self-action and interaction sequences. We hypothesize that the cerebellum predicts how actions by the self and other people will be executed, what our most likely responses are to these actions, and what the typical sequence of these actions is. This function of forward controller allows people to anticipate, predict and understand actions by the self or other persons and their consequences for the self, *to automatize these inferences for intuitive and rapid execution* [italics added], and to instantly detect disruptions in action sequences. These are important social functions. Consequently, if neurological disorders affect the cerebellum, detrimental effects on social functionality might be found, especially on more complex and abstract social cognitive processes. The cerebellum would be a “forward controller” that not only constructs and predicts motor sequences, but also takes part in the construction of internal models that support social and self-cognition. In this respect, the cerebellum crucially adds to the fluent understanding of planned and observed social inter-actions and contributes to sequencing mechanisms that organize autobiographical knowledge. (p. 35).

For the purposes here, these cerebellar forward controller models represent the learning of sequences of social interaction whether they be everyday cultural interactions, the one-on-one social learning of precise stone-tool knapping (stone shaping), or a child learning of arithmetic in a one-on-one tutoring situation. These forward controller processes suggest that the learning of automatization of intuitive execution of complex and abstract process, as in a child’s successfully learning the *automaticity and fluidity* of number sense in arithmetic [46], might be adversely affected by disorders of the cerebellum (e.g., the MLD’s described earlier in [19]). These points comport completely with Vandervert’s [10] arguments on the role of the cerebellum in inner speech driven automaticity in the learning of number sense (the automatic-intuitive manipulation of numbers) and dyscalculia.

## Conclusions

Within the framework of the evolution of the social cerebellum it is concluded that mathematics (the science of patterns) can be argued to have social origins within the evolution of stone-tool making. It is argued that this evolution selected toward a social cognitive cerebellum which arose from the arduous, repetitive precision knapping (stone shaping) requirements that over a period of a million-plus years selected from mentalizing the kinematics and dynamics as observed and modeled in Theory of Mind (ToM) of more experienced stone knappers. Thus, the patterns of sequences in stone-tool making and the patterns that constitute mathematics may be seen as homological (sharing common evolutionary descent). Specifically, through composition, decomposition and blending of cerebellar internal models the patterns of sequences associated with these kinematics and kinematics became consciously applicable, through autobiographical abstraction in inner speech and language evolution, to all kinematics and dynamics as patterns of number sequences. This social origin of mathematics can be applied to strategies for the optimization of one-on-one arithmetic tutoring of children with mathematical learning disabilities (MLD’s) including dyscalculia.

## Data Availability

NA

## Footnotes

1 Lee Arthur Steen, while Chairman of the Conference Board of the Mathematical Sciences in the United States, defined mathematics as “the science of patterns:” Mathematics is the science of patterns. The mathematician seeks patterns in number, in space, in science, in computers, and in imagination. Mathematical theories explain the relations among patterns; functions and maps, operators and morphisms bind one type of pattern to another to yield lasting mathematical structures. Applications of mathematics use these patterns to “explain” and predict natural phenomena that fit the patterns. (1988, p. 616).

2 This view does not necessarily contradict Overmann’s [18] work on the material origins of number. Rather, it can augment her work with verifiable socially mediated cerebellar mechanisms behind the evolution of mathematics.

3 Vandervert (2015) and Vandervert and Vandervert-Moe [36] argued that such compositions and blends are often sent to working memory areas of the cerebral cortex in a spontaneous fashion and thus give rise to the intuitive insight behind creativity. With the evolution of the cerebellum of *Homo sapiens* (Leiner, Leiner & Dow, 1986, 1989), Vandervert [31, 37] argued that this process was prominent (perhaps predominant) in driving the evolution of culture.
